# Complement System Proteins in the Human Aqueous Humor and Their Association with Primary Open-Angle Glaucoma

**DOI:** 10.3390/jpm13091400

**Published:** 2023-09-19

**Authors:** Ayushi Vashishtha, Sharon W. Maina, Jeremy Altman, Garrett Jones, Tae Jin Lee, Kathryn E. Bollinger, Lane Ulrich, Marc Töteberg-Harms, Amy J. Estes, Wenbo Zhi, Shruti Sharma, Ashok Sharma

**Affiliations:** 1 Morsani College of Medicine, University of South Florida, Tampa, FL 33612, USA; ayushiv@usf.edu; 2 Center for Biotechnology and Genomic Medicine, Medical College of Georgia, Augusta University, Augusta, GA 30912, USA; smaina@augusta.edu (S.W.M.); jaltman@augusta.edu (J.A.); garjones@augusta.edu (G.J.); talee@augusta.edu (T.J.L.); wzhi@augusta.edu (W.Z.); shsharma@augusta.edu (S.S.); 3 Department of Ophthalmology, Medical College of Georgia, Augusta University, Augusta, GA 30912, USA; kbollinger@augusta.edu (K.E.B.); lulrich@augusta.edu (L.U.); mtoetebergharms@augusta.edu (M.T.-H.); aestes@augusta.edu (A.J.E.); 4 Department of Population Health Sciences, Medical College of Georgia, Augusta University, Augusta, GA 30912, USA

**Keywords:** glaucoma, POAG, complement proteins, aqueous humor, proteomics, mass spectrometry

## Abstract

This study discovers the complement protein profile in the aqueous humor (AH) of human subjects and investigates its association with primary open-angle glaucoma (POAG) pathogenesis. Among the 32 complement proteins identified, 22 were highly abundant and detected in more than 50% of AH samples. The most predominant active complement proteins in the AH are C3, C4B, C4A, CFB, CFD, and C9. Additionally, the most prevalent complement regulators and receptors include CLU, SERPING1, F2, CFH, CFI, and VTN. Significant alterations in complement proteins were observed in individuals with POAG compared to those with cataracts. Specifically, complement protein F2 was upregulated, while C8G, C6, and CFH were downregulated in POAG samples. Stratification of the samples by race and sex revealed distinct alterations of complement proteins in patients with POAG. In the African American cohort, five complement proteins (C4A, C4B, F2, C7, and C3) were upregulated in POAG compared to cataract patients. In the Caucasian cohort, eight complement proteins (C3, SERPING1, CFI, CLU, CFHR1, C8G, C6, and CFH) were downregulated in the POAG samples compared to the cataract samples. Within the male cohort, three complement proteins (CLU, C6, and CFH) were downregulated in POAG patients compared to those with cataracts. Whereas, within the female cohort, two complement proteins (C4B and F2) were upregulated and one (C8G) downregulated in the POAG samples when compared to cataracts. Discerning these changes in the AH complement protein profile will assist in the development of tailored therapies to modulate the complement system for managing ocular disorders. These insights may also lead to novel biomarkers for diagnosing and monitoring disease progression.

## 1. Introduction

The ocular environment exhibits a unique combination of immunosuppressive and anti-inflammatory characteristics referred to as ocular immune privilege [1,2]. Several mechanisms contribute to maintaining this privilege, including mucosal immunity [3,4], blood–ocular barriers [5,6,7,8], ocular parenchymal cells [9], and the secretion of regulatory molecules in the vitreous and aqueous humor [10,11]. However, disruption in any of these pathways can disturb the ocular immune privilege, leading to the development of ocular pathophysiological conditions [12,13,14,15,16].

The aqueous humor (AH) serves various vital functions, including the regulation of intraocular pressure (IOP), the removal of metabolic waste, and the supply of nutrients and oxygen to non-vascular ocular tissues [17]. The AH also contains a significant concentration of immunomodulatory molecules [17], including the prominent complement family [18].

Complement proteins are involved in various host defense and inflammatory responses, playing an essential role in immune modulation within the eye [19,20,21]. Previous studies have shown that alterations in complement proteins within the AH are associated with the pathogenesis of various ocular disorders, including primary open-angle glaucoma (POAG), uveitis, and macular degeneration [22,23,24]. Additionally, inhibiting components of the complement cascade have shown promise in preventing synaptic degeneration in experimental models of glaucoma [25,26].

The objective of this study is to investigate the presence of complement proteins in the AH of human subjects and examine their association with POAG pathogenesis. In this study, we quantified the levels of complement proteins in the AH of 258 subjects who underwent either glaucoma or cataract surgeries. Furthermore, we evaluated the association of these proteins with POAG, considering potential differences based on sex and race.

## 2. Materials and Methods

### 2.1. Sample Collection

Ethical approval for this study was obtained from the Institutional Review Board of Augusta University (IRB #611480), and written informed consent was acquired from all participants prior to sample collection. Subjects undergoing cataract or glaucoma surgery were included. During the surgical procedure, after making the initial corneal incision, ~60 μL AH was aspirated from the anterior chamber using a cortical cleaving hydrodissector (Beaver-Visitec International #585157). The samples were carefully collected in sterile tubes and immediately frozen at −80 °C. No additional ocular procedures or modifications were performed during the AH collection process. The study included 196 AH samples from cataract surgery patients and 62 AH samples from POAG patients, as outlined in Table 1. Additional patient information is available in Supplementary File S1. 

### 2.2. Liquid Chromatography–Tandem Mass Spectrometry Analysis

The AH samples (60 μL) were subjected to lyophilization and then reconstituted in 30 μL of 8 M urea in 50 mM Tris-HCl (pH 8). Cysteine residues were reduced using 20 mM dithiothreitol and alkylated with 55 mM iodoacetamide. Subsequently, 240 μL of a 50 mM ammonium bicarbonate buffer was added to reduce the urea concentration to less than 1 mM. The protein concentration was determined using a Bradford assay kit (Pierce, Rockford, IL, USA) following the manufacturer’s instructions. Protein digestion was performed by adding trypsin at a 1:20 ratio (*w*/*w*) and incubating overnight at 37 °C.

The digested AH samples were purified using a C18 spin plate (Nest Group, Southborough, MA, USA), and the resulting peptides were separated using an Ultimate 3000 nano-UPLC system (Thermo Fisher Scientific, Waltham, MA, USA) and analyzed using an Orbitrap Fusion Tribrid mass spectrometer (Thermo Fisher Scientific, Waltham, MA, USA). A 6 μL portion of the reconstituted peptide mixture was trapped and washed on a Pepmap100 C18 trap (5 μm, 0.3 × 5 mm) at a flow rate of 20 μL/min using a solution of 2% acetonitrile in water with 0.1% formic acid for 10 min. The peptides were then separated on a Pepman100 RSLC C18 column (2.0 μm, 75 μm × 150 mm) using a gradient of 2% to 40% acetonitrile with 0.1% formic acid over 120 min (flow rate: 300 nL/min; column temperature: 40 °C). Eluted peptides were subjected to data-dependent acquisition in positive mode with the following settings: precursor scan in the Orbitrap MS analyzer at 120,000 FWHM from 300 to 1500 *m*/*z*, and MS/MS scans in top speed mode (2-s cycle time) using the ion-trap MS analyzer. Dynamic exclusion settings were applied (repeat count: 1; repeat duration: 15 s; exclusion duration: 30 s). Higher-energy C-trap dissociation (HCD) was employed as the fragmentation method with a normalized collision energy of 30%.

### 2.3. Protein Identification and Quantification

The raw MS data were processed using Proteome Discoverer software (v1.4; Thermo Fisher Scientific, Waltham, MA, USA) and analyzed with the SequestHT algorithm. The search was performed against the Uniprot-SwissProt human database, which contained 20,385 entries. The search parameters were set as follows: 10 ppm precursor ion tolerance and 0.6 Da product ion tolerance; static carbidomethylation (+57.021 Da) for cysteine; dynamic oxidation (+15.995 Da) for methionine; and dynamic phosphorylation (+79.966 Da) for serine, threonine, and tyrosine.

To validate peptide spectrum matches (PSMs), the Percolator peptide spectrum matching validator within the Proteome Discoverer software was utilized. Proteins with similar peptide compositions that could not be differentiated based on MS/MS analysis alone were grouped using parsimony principles. Spectrum counts (number of PSMs) and identities for each protein were then exported as a semi-quantitative measure of the relative protein levels detected in each AH sample.

### 2.4. Optical Coherence Tomography (OCT) and Heidelberg Retinal Tomography (HRT) Measurements

Retinal nerve fiber layer (RNFL) thickness was measured using SPECTRALIS Tracking Laser Tomography (Heidelberg Engineering, Heidelberg, Germany). This system captured a 24-line high-resolution radial scan, 3.5 mm in diameter, of the optic nerve head centered on Bruch’s membrane opening (BMO). The region between the BMO and the closest point on the internal limiting membrane was defined as the “neuroretinal rim”. Three circular scans centered on the optic nerve head, as delineated by the BMO, were used to determine RNFL thickness. The results were compared to a reference database of healthy eyes, adjusted for BMO size and age. The findings were presented in the Garway–Heath sector format, facilitating structural and functional correlations. In addition, the ganglion cell layer (GCL) and the macula were evaluated using multi-layer segmentation software, and the results were visualized through a GCL thickness map.

The Heidelberg Retinal Tomograph (Heidelberg Engineering, Heidelberg, Germany) was also employed for imaging the optic nerve head. Multiple 3D images were captured at different depths, providing a comprehensive representation of the optic nerve and surrounding retina. These images were combined to generate a 3D representation of the entire optic nerve, from which various stereometric parameters were calculated. These parameters include the cup shape measure, cup-to-disc area ratio, and rim volume. The cup shape measure quantifies the configuration of the cup, incorporating assessments of cup wall steepness and depth variation. In individuals without glaucomatous damage, this measure is generally negative. Conversely, glaucomatous eyes typically exhibit values that are less negative or positive [27]. The cup-to-disc area ratio assesses the extent of cupping relative to the area of the optic disc. In patients with uncontrolled glaucoma, this ratio tends to increase over time. Lastly, the rim area assesses the neuroretinal rim, which marks the boundary between the cup and the disc. Among patients with glaucoma, this metric is typically lower, indicating the degradation or loss of nerve fibers within the neuroretinal rim.

### 2.5. Statistical Analyses

Statistical analyses were conducted using the R project for Statistical Computing (version 3.6.3). The chi-square test and two-tailed *t*-test were used to compare the clinical characteristics of POAG and cataract patients. Prior to analysis, the peptide spectrum matching (PSM) values obtained from the LC-MS/MS analysis were quantile normalized, and the proportion of samples in which each protein was detected was determined. Disease-specific differences in complement protein levels were assessed using a negative binomial regression model. Spearman’s correlation coefficients were employed to evaluate the correlations between complement proteins and clinical parameters, such as OCT and HRT measures. The significance level was set at *p* < 0.05.

## 3. Results

### 3.1. Complement Proteins Detected in the Human Aqueous Humor

**Figure 1:**
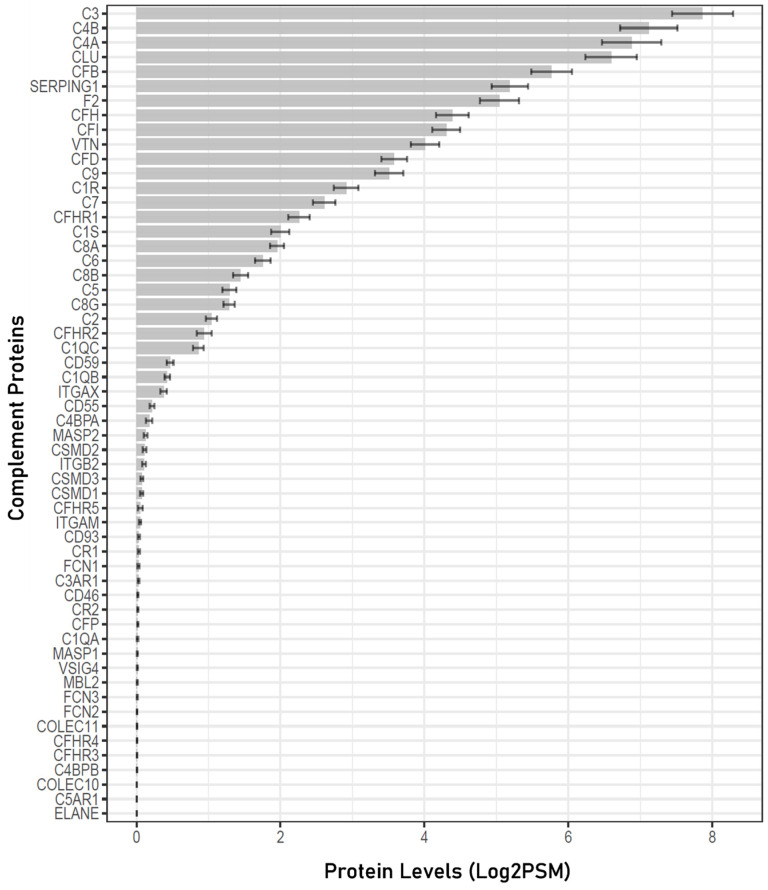
Complement proteins detected in the human aqueous humor. A total of 32 complement proteins were detected in the human AH samples analyzed. The mean PSM values were log2 transformed for comparison.

According to the HUGO Gene Nomenclature Committee, there are a total of 56 proteins that belong to the complement system group, including 27 complement system activation components and 29 complement system regulators and receptors (https://www.genenames.org; accessed on 17 July 2023) [28]. In the 258 human aqueous humor samples analyzed, 32 out of the 56 complement proteins known in the human proteome were detected (Figure 1). Complete data files are available in Supplementary File S2.

The mean PSM values and the proportion of samples in which each specific complement protein was detected are provided in Table 2. Among these proteins, 22 were detected in more than 50% of the AH samples, while 6 were detected in all 258 samples. The most abundant complement proteins identified were as follows, listed with their respective mean PSM values ± standard deviation (SD): complement C3 (227.59 ± 68.1), complement C4B (134.07 ± 64.43), complement C4A (112.39 ± 77.95), clusterin (CLU) (95.22 ± 40.97), and complement factor B (CFB) (52.12 ± 13.43) (Table 2). 

### 3.2. Alterations in Aqueous Humor Complement Proteins Associated with POAG

**Figure 2:**
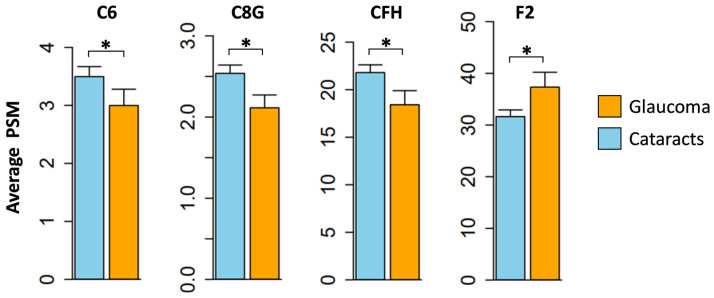
Alterations in the aqueous humor complement proteins associated with glaucoma. Fold change values were compared across groups (POAG vs. cataracts). A total of four proteins (C6, C8G, CFH, and F2) displayed significant alterations in protein levels. * *p* < 0.05.

The specific changes in complement proteins within the aqueous humor of POAG patients were examined (Figure 2; Table 3). The fold change (FC) values indicate the alterations in protein levels observed in the POAG group (n = 62) compared to the cataract group (n = 196). The results revealed decreased levels of complement component C8 gamma chain (C8G) (FC = 0.83, *p* = 0.026), complement C6 (FC = 0.81, *p* = 0.032), and complement factor H (CFH) (FC = 0.77, *p* = 0.024) in POAG subjects relative to those with cataracts. Conversely, the expression of prothrombin (F2) (FC = 1.19, *p* = 0.046) was significantly higher in the POAG group compared to the cataract cohort. 

### 3.3. Race-Specific Alterations in Aqueous Humor Complement Proteins Associated with POAG

**Figure 3:**
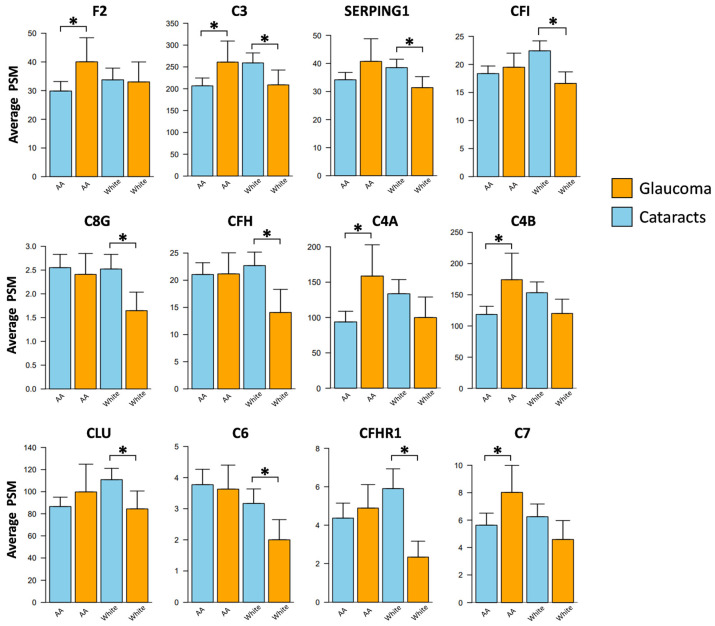
Race-specific alterations in the aqueous humor complement proteins associated with glaucoma. Patient samples were stratified according to race and compared across the two disease cohorts. Five proteins were found significantly altered within African Americans and eight within white subjects between the POAG and cataract cohorts. AA = Black/African American subjects; * *p* < 0.05.

After stratifying the samples based on race, eleven complement proteins exhibited significant alterations in POAG patients compared to cataract subjects (Figure 3; Table 3). In African Americans with POAG, five complement proteins were significantly elevated in the AH samples compared to those with cataracts: C4A (FC = 2.33, *p* = 0.027), C4B (FC = 1.69, *p* = 0.015), complement component C7 (FC = 1.38, *p* = 0.025), F2 (FC = 1.31, *p* = 0.023), and C3 (FC = 1.21, *p* = 0.027). No proteins were found significantly decreased in African American AH samples with POAG compared to cataracts.

In the Caucasian cohort, eight complement proteins were found to be significantly lower in the POAG group compared to cataract subjects: C3 (FC = 0.82, *p* = 0.038), plasma protease C1 inhibitor (SERPING1) (FC = 0.83, *p* = 0.015), complement factor I (CFI) (FC = 0.77, *p* = 0.007), CLU (FC = 0.77, *p* = 0.021), complement factor H-related protein 1 (CFHR1) (FC = 0.48, *p* = 0.002), C8G (FC = 0.67, *p* = 0.004), C6 (FC = 0.63, *p* = 0.002), and CFH (FC = 0.57, *p* = 0.003). Interestingly, within the Caucasian cohort, no complement proteins were found to be elevated in glaucoma samples compared to cataracts. These race-specific differences in complement proteins between POAG and cataracts are shown in Figure 3.

### 3.4. Sex-Specific Alterations in the Aqueous Humor Complement Proteins Associated with POAG 

**Figure 4:**
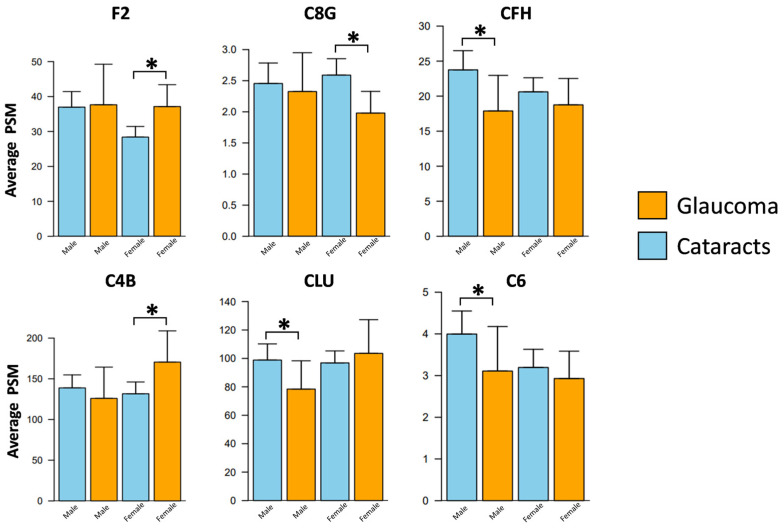
Sex-specific alterations in the aqueous humor complement proteins associated with glaucoma. Patient samples were stratified according to sex and compared across the two disease cohorts. Three proteins were found significantly altered within males and three within females between the POAG and cataract subjects. * *p* < 0.05.

After stratifying the samples by sex, we identified significant differences in six complement proteins between the two patient groups (Figure 4; Table 4). Among males, the POAG group exhibited significant decreases in three complement proteins compared to the cataract group: CLU (FC = 0.78, *p* = 0.046), C6 (FC = 0.67, *p* = 0.009), and CFH (FC = 0.63, *p* = 0.015). Conversely, no significant increases in complement proteins were observed in the POAG group relative to cataracts within the male cohort. In contrast, within the female samples, two complement proteins showed significant upregulation in the POAG group compared to cataracts: C4B (FC = 1.56, *p* = 0.030) and F2 (FC = 1.37, *p* = 0.007). Additionally, C8G (FC = 0.78, *p* = 0.025) exhibited downregulation in the POAG group compared to cataracts (Figure 4).

### 3.5. Alterations in Complement Proteins Correlated with Pattern Standard Deviation (PSD) and Visual Field Index (VFI)

Two glaucoma metrics, PSD and VFI, have been correlated with disease severity. To determine if any complement proteins within the AH were associated with these metrics, a correlation analysis was conducted within the POAG cohort (Table 5). The majority of the correlation coefficients (R) did not exceed the absolute value of 0.5, indicating that the correlations, whether positive or negative, were not strong (|R| > 0.5) [29]. Seven complement proteins demonstrated a significant, positive correlation with PSD, with |R| values ranging from 0.33 to 0.47. These proteins include C5 (R = 0.47, *p* = 0.002), C7 (R = 0.43, *p* = 0.006), complement C1R (R = 0.39, *p* = 0.014), C4B (R = 0.38, *p* = 0.019), complement component C8 alpha chain (C8A) (R = 0.37, *p* = 0.020), F2 (R = 0.36, *p* = 0.024), and C4A (R = 0.33, *p* = 0.038). For the VFI, seven complement proteins including C7 (R = −0.64, *p* = 0.0006), C8G (R = −0.54, *p* = 0.006), C1R (R = −0.52, *p* = 0.008), F2 (R = −0.49, *p* = 0.014), C4B (R = −0.46, *p* = 0.020), C4A (R = −0.44, *p* = 0.027), and C9 (R = −0.44, *p* = 0.027) demonstrated a significant negative correlation.

### 3.6. Complement Proteins Correlated with Ocular Stereometric Heidelberg Retinal Tomography (HRT) Parameters

Complement proteins were examined in relation to ocular stereometric HRT parameters. Moderate correlations (0.5 > |R| ≥ 0.3) and weak correlations (0.30 > |R| ≥ 0.20) were observed between several complement proteins and specific stereometric patterns, including cup area, cup volume, cup depth, disc area, and cup-to-disc (CD) ratio (Table 6). Specifically, six complement proteins (CFI, VTN, CLU, C3, CFH, and CFHR1) exhibited negative correlations with cup area. The |R| values for these proteins ranged from 0.17 to 0.33. For cup volume, nine complement proteins (CFI, VTN, C3, CLU, CFH, CFHR1, C4B, C2, and F2) demonstrated negative correlations, with |R| values ranging from 0.16 to 0.33. Regarding maximum cup depth, eight complement proteins (CFI, VTN, C3, CFH, F2, CFHR1, C5, and C8B) were found to be negatively correlated, with |R| values ranging from 0.17 to 0.27. Mean cup depth found four proteins (CFI, CFH, C3, and CFHR1) with significant correction. Of these, all displayed negative R values, with |R| values varying from 0.17 to 0.23. For the disc area, four complement proteins (CFI, VTN, CFHR1, and C3) demonstrated a negative correlation with the metric, and |R| values ranged from 0.17 to 0.19. Furthermore, six complement proteins (CFI, CLU, CFH, VTN, C3, and CFHR1) exhibited a negative correlation with CD area ratio, with |R| values varying from 0.16 to 0.28.

## 4. Discussion

Complement proteins play a critical role in immune responses and inflammatory processes, with their dysregulation being linked to various ocular disorders, including glaucoma. This study provides a comprehensive analysis of the complement proteins present in the human AH. In addition, we explored the relationship between AH complement protein profiles and primary open-angle glaucoma (POAG). Liquid chromatography–tandem mass spectrometry (LC-MS/MS) was utilized to both identify and quantify complement proteins in the AH samples collected from both POAG and cataract patients. 

The findings reveal significant changes in complement proteins among POAG patients compared to those with cataracts. Furthermore, these alterations exhibit variations based on race and sex. Gaining insight into complement protein profiles in the AH will assist us in the development of targeted therapies to effectively modulate the complement system for treating ocular disorders and establish novel biomarkers for diagnosing and monitoring disease progression.

We successfully identified a total of 32 complement proteins within the AH samples collected from 62 POAG and 196 cataract patients. Twenty-two were notably abundant, appearing in more than 50% of the AH samples and corroborating previous findings [30,31]. Proteomic analysis revealed significant downregulation of three complement proteins, including C6, C8G, and CFH, while one protein, F2, was upregulated in POAG subjects compared to cataract controls.

Complement proteins trigger the formation of the terminal membrane attack complex, (MAC), which plays a crucial role in targeting pathogens and regulating immune responses [32,33]. The downregulation of C6 in the AH from POAG subjects is intriguing, as past reports have found upregulated MAC components, such as C6, in human and mouse glaucomatous retinas [34]. Lower C6 levels may be the body’s attempt to regulate or compensate for increased oxidative stress, apoptosis, or dysregulation in immune responses. Moreover, upon binding to the α-chain of C5b, C6 gains the capacity to interact with cell membranes [35]. The greater activation of the complement cascades in POAG patients might lead to the conversation of C6 and subsequent association to target cell membranes, resulting in reduced levels within the measured AH. Alternatively, recent studies highlighting independent complement gene transcription and protein synthesis within retinal cells offer an alternative approach for decreased AH C6 levels [36,37,38]. The elevated cell death in the retinal cells of POAG patients could contribute to a decrease in C6 levels in the surrounding environment. Nevertheless, investigating the origins of complement proteins within the AH and their correlation to retinal protein profiles could provide further insight. 

Another component of the MAC, C8G [39], was found significantly downregulated in AH from POAG subjects compared to cataracts. Though the role of C8G outside of MAC formation is limited, one study found C8 subunit deficiency associated with recurrent Neisserial bacterial infections in humans [40]. Also, recent studies have shown the neuroprotective effects of C8G and its anti-inflammatory properties within the brain, where the upregulation of C8G acts as an antagonist of microglial S1PR2, resulting in the attenuation of neuroinflammation in mice models [41,42,43]. A decrease in C8G levels in the AH of POAG subjects could signal impairment in anti-inflammatory pathways and increased risk for infection compared to non-glaucoma individuals. 

As an essential regulator, CFH indirectly inhibits complement pathway elements like C3b, C3 convertase, and subsequently, MAC formation [44,45]. Prior studies have reported the downregulation of CFH in retinas from glaucomatous patients and mice exposed to oxidative stress [34,46,47]. The observed reduction in CFH levels in the retina of glaucoma patients aligns with our findings and may suggest a potential AH biomarker for elevated oxidative stress and POAG. However, it is notable that decreased CFH levels in the AH of the POAG patients coincide with a decreased MAC component, C6. As CFH mainly inhibits the alternative pathway [48], a corresponding decrease in C6 may signal downstream pathway dysfunction or other active mechanisms hindering C6 accumulation. This may also suggest a non-MAC-associated role for CFH in POAG. However, further studies on the regulatory influence of CFH on MAC regulation and POAG in the AH are needed. 

In our study, we also found elevated prothrombin (F2) in POAG aqueous humor samples. It is primarily recognized for its role in the coagulation cascade [49]. The pathogenesis of glaucoma involves vascular dysregulation and the associated oxidative stress [43,50]. Changes in the coagulation system, including increased F2 levels, could contribute to microvascular abnormalities or blood flow changes in the optic nerve head and retina that may ultimately contribute to retinal cell damage in POAG patients. Consistent with our findings of elevated F2 levels in the AH samples, another study reported an increase in F2 concentrations in the venous blood of POAG patients when compared to the controls and normal pressure glaucoma patients [51], which might suggest a potential involvement of F2 in the pathophysiology of POAG.

Interestingly, complement C3 was the highest detected protein in our AH samples. Furthermore, C3 levels increased 1.21-fold in African American POAG patients compared to those with cataracts, whereas decreased levels were detected in Caucasians with POAG, suggesting that there is perhaps a race-dependent role of the alternative complement pathway in glaucoma. Several studies have consistently reported a higher prevalence of glaucoma within the African American population, although the specific factors contributing to this disparity remain unclear [52]. Upregulated C3 levels in the AH of African Americans could be one of the factors posing higher risks for glaucoma severity, with subsequent complement cascade activation triggering the proteolytic cleavage of C3 into C3a and C4b. C3a and C5a are anaphylatoxins that have been linked to glaucoma pathogenesis [53,54,55] and the progression of other ocular diseases, like AMD [56]. As potential inflammatory mediators, these molecules can target a broad range of immune and non-immune cells, causing an increase in the permeability of small blood vessels, oxidative bursts, and the release of histamine [57]. Controlling the regulation of these molecules offers the potential to reduce the severity of glaucoma, and anti-C3a and anti-C5a drugs are currently in clinical trials as treatment options for late-stage AMD [58]. 

Furthermore, the findings from our study indicate that four additional complement proteins, C4A, C4B, F2, and C7, were significantly elevated in the African American population with glaucoma compared to those with cataracts. However, eight proteins (C6, C8G, CFH, C3, CFHR1, CFI, CLU, and SERPING1) were significantly downregulated in Caucasian glaucoma subjects. These findings indicate race-specific differences in complement protein profiles associated with glaucoma, suggesting potential variations in the underlying pathogenesis and immune response between the two racial groups. Cleavage of C4 into C4A and C4B is known to generate classical pathway C3 convertase [59,60], an enzyme that cleaves C3 into C3A and C3B, leading to a potential elevation in pro-inflammatory pathways. The increased levels of AH C4A and C4B in African American POAG subjects compared to those with cataracts emphasize the potential involvement of the complement cascade in the accelerated onset and progression of glaucoma within this demographic group. 

Within the Caucasian cohort, several regulatory proteins, specifically CFI and CLU, were also downregulated in POAG. CFI is a serine protease that inhibits the complement pathways through the degradation of activated complement proteins C3A and C4B [61]. CLU has been investigated for its potential neuroprotective effects [62] and acts as an extracellular molecular chaperone [63,64], where it can capture and clear soluble precursors to the membrane attack complex. With its involvement in the removal of potentially toxic protein oligomers, downregulation in POAG patients compared to the controls may contribute to the buildup of inflammatory molecules within the ocular environment.

Further, in our study, we found sex-specific differences, with the levels of CLU, C6, and CFH proteins significantly decreased in males with POAG compared to the controls. As an inhibitor of MAC formation, reduced CLU in males could render retinal neurons more vulnerable to complement-mediated inflammation and cell damage. Further, decreased levels of CFH might suggest a compromised regulatory control over the alternative complement pathway. Complement proteins C4B and F2 increased while C8G decreased in the AH of female POAG subjects. Increased C4B levels may signify heightened activation of the classical and lectin pathways of the complement system, while increased F2 in the AH may hint at broader implications in the vasculature of the eye [65]. A previous study has shown that inhibition of the classical pathway of the complement cascade prevents early dendritic and synaptic degeneration in mouse and rat models of glaucoma [66].

While these findings expand our understandings of complement proteins in the AH and their relation to POAG, it should be noted that there were several limitations to our study. We were unable to compare African American males/females to Caucasian males/females with POAG and cataracts due to limited statistical power. Another limitation is the potential influence of anti-glaucoma therapy on the AH complement profile. Given that certain anti-glaucoma treatments have been reported to induce alterations in the dynamics of AH production [67], there is potential for these therapies to influence the abundance and activity of complement proteins within the ocular environment. However, since most patients undergoing glaucoma surgery are already receiving anti-glaucoma therapy, the absence of a substantial treatment-naïve cohort constrained our statistical power. Therefore, a detailed examination focused on the interplay between anti-glaucoma therapies and complement protein expression within the AH is necessary for a more complete understanding. Furthermore, while alterations in the complement composition of the AH may be associated with POAG, more studies of the optical nerve and retinal tissues in relation to the AH are needed to better understand the molecular mechanisms regulating POAG.

## 5. Conclusions

In summary, this study uses modern LC-MS/MS analyses to advance our current knowledge of aqueous humor-associated complement proteins in relation to POAG. Our results provide insights into the relation between AH’s complement profile and its differential expression within race, sex, and glaucoma subgroups. Further investigations are needed to unveil the precise contribution of the complement system to glaucoma disease pathology and its impact on ocular susceptibility. Future research incorporating complement proteins in ocular diseases will help advance personalized treatment strategies, ultimately improving glaucoma management and therapies. 

## Figures and Tables

**Table 1:** Demographic information of the subjects.

Demographic Characteristic	Cataract (n = 196)	POAG (n = 62)	*p*-Value
Age (years, mean ± SD)	67.38 ± 9.48	67.87 ± 11.23	0.76 ^a^
Sex (F/M)	122/74	38/24	1 ^b^
Race (African Americans/Caucasians)	107/89	38/24	0.44 ^b^

a: two-sample *t*-test b: chi-square test.

**Table 2:** Complement system proteins detected in the human aqueous humor.

UniProt ID	Gene Symbol	Description	Mean PSM Value (±SD)	Proportion of Samples Detected (%)
		Activation Components		
P01024	C3	Complement C3	227.59 ± 68.1	100.0
P0C0L5	C4B	Complement C4B	134.07 ± 64.43	96.5
P0C0L4	C4A	Complement C4-A	112.39 ± 77.95	79.8
P00751	CFB	Complement factor B	52.12 ± 13.43	100
P00746	CFD	Complement factor D	11.32 ± 6.3	99.2
P02748	C9	Complement component C9	11.03 ± 8.76	93.8
P00736	C1R	Complement C1r subcomponent	6.92 ± 5.76	85.7
P10643	C7	Complement component C7	5.14 ± 4.49	91.5
P09871	C1S	Complement C1s subcomponent	3.26 ± 3.33	76.4
P07357	C8A	Complement component C8 alpha chain	3.13 ± 2.59	90.3
P13671	C6	Complement component C6	2.57 ± 2.72	74.8
P07358	C8B	Complement component C8 beta chain	1.91 ± 2.62	54.7
P07360	C8G	Complement component C8 gamma chain	1.58 ± 1.63	65.9
P01031	C5	Complement C5	1.55 ± 2.16	58.9
P06681	C2	Complement C2	1.11 ± 1.43	54.7
P02747	C1QC	Complement C1q subcomponent subunit C	0.81 ± 1.2	43.0
P02746	C1QB	Complement C1q subcomponent subunit B	0.36 ± 0.6	29.8
O00187	MASP2	Mannan-binding lectin serine protease 2	0.09 ± 0.31	8.1
O00602	FCN1	Ficolin-1	ND	ND
P48740	MASP1	Mannan-binding lectin serine protease 1	ND	ND
P02745	C1QA	Complement C1q subcomponent subunit A	ND	ND
P27918	CFP	Properdin	ND	ND
O75636	FCN3	Ficolin-3	ND	ND
P11226	MBL2	Mannose-binding protein C	ND	ND
Q9BWP8	COLEC11	Collectin subfamily member 11	ND	ND
Q15485	FCN2	Ficolin-2	ND	ND
Q9Y6Z7	COLEC10	Collectin subfamily member 10	ND	ND
		Regulators and Receptors		
P10909	CLU	Clusterin	95.22 ± 40.97	100.0
P05155	SERPING1	Plasma protease C1 inhibitor	35.57 ± 12.07	100.0
P00734	F2	Prothrombin	31.86 ± 15.52	100.0
P08603	CFH	Complement factor H	21.11 ± 12.14	98.1
P05156	CFI	Complement factor I	19.12 ± 7.18	100.0
P04004	VTN	Vitronectin	14.92 ± 6.39	98.1
Q03591	CFHR1	Complement factor H-related protein 1	3.85 ± 4.28	54.7
P36980	CFHR2	Complement factor H-related protein 2	0.86 ± 1.98	20.9
P13987	CD59	CD59 glycoprotein	0.38 ± 0.7	29.1
P20702	ITGAX	Integrin alpha-X	0.31 ± 0.68	20.5
P08174	CD55	Complement decay-accelerating factor	0.17 ± 0.48	13.6
P04003	C4BPA	C4b-binding protein alpha chain	0.12 ± 0.64	6.6
Q7Z408	CSMD2	CUB and sushi domain-containing protein 2	0.08 ± 0.3	6.6
P05107	ITGB2	Integrin beta-2	0.07 ± 0.3	6.2
Q7Z407	CSMD3	CUB and sushi domain-containing protein 3	ND	ND
Q96PZ7	CSMD1	CUB and sushi domain-containing protein 1	ND	ND
P11215	ITGAM	Integrin alpha-M	ND	ND
Q9BXR6	CFHR5	Complement factor H-related protein 5	ND	ND
P17927	CR1	Complement receptor type 1	ND	ND
Q9NPY3	CD93	Complement component C1q receptor	ND	ND
Q16581	C3AR1	C3a anaphylatoxin chemotactic receptor	ND	ND
P20023	CR2	Complement receptor type 2	ND	ND
P15529	CD46	Membrane cofactor protein	ND	ND
Q9Y279	VSIG4	V-set and immunoglobulin domain-containing protein 4	ND	ND
P20851	C4BPB	C4b-binding protein beta chain	ND	ND
Q92496	CFHR4	Complement factor H-related protein 4	ND	ND
Q02985	CFHR3	Complement factor H-related protein 3	ND	ND
P08246	ELANE	Neutrophil elastase	ND	ND
P21730	C5AR1	Complement C5a receptor 1	ND	ND

ND: not detected; PSM: peptide spectrum match.

**Table 3:** Race-specific changes in the aqueous humor complement proteins in glaucoma patients.

Gene Symbol	Description	Overall		African American		Caucasian	
		FC	*p*-Value	FC	*p*-Value	FC	*p*-Value
C4A	Complement C4-A	1.44	0.210	2.33	0.027 *	0.72	0.452
C4B	Complement C4B	1.30	0.074	1.69	0.015 *	0.89	0.517
F2	Prothrombin	1.19	0.046 *	1.31	0.023 *	1.03	0.811
C7	Complement component C7	1.06	0.576	1.38	0.025 *	0.72	0.057
C3	Complement C3	1.03	0.607	1.21	0.027 *	0.82	0.038 *
SERPING1	Plasma protease C1 inhibitor	1.00	0.946	1.13	0.114	0.83	0.015 *
CFI	Complement factor I	0.93	0.261	1.06	0.490	0.77	0.007 *
CLU	Clusterin	0.93	0.325	1.05	0.626	0.77	0.021 *
CFHR1	Complement factor H-related protein 1	0.85	0.273	1.25	0.257	0.48	0.002 *
C8G	Complement component C8 gamma chain	0.83	0.026 *	0.94	0.600	0.67	0.004 *
C6	Complement component C6	0.81	0.032 *	0.95	0.716	0.63	0.002 *
CFH	Complement factor H	0.77	0.024 *	0.95	0.702	0.57	0.003 *

FC: fold change in glaucoma patients compared to cataract patients; * *p* < 0.05.

**Table 4:** Sex-specific changes in the aqueous humor complement proteins in glaucoma patients.

Gene Symbol	Description	Overall		Male		Female	
		FC	*p*-Value	FC	*p*-Value	FC	*p*-Value
C4B	Complement C4B	1.30	0.074	0.96	0.853	1.56	0.030 *
F2	Prothrombin	1.19	0.046 *	0.94	0.630	1.37	0.007 *
CLU	Clusterin	0.93	0.325	0.78	0.046 *	1.03	0.781
C8G	Complement component C8 gamma chain	0.83	0.026 *	0.88	0.388	0.78	0.025 *
C6	Complement component C6	0.81	0.032 *	0.67	0.009 *	0.92	0.519
CFH	Complement factor H	0.77	0.024 *	0.63	0.015 *	0.87	0.339

FC: fold change in glaucoma patients compared to cataract patients; * *p* < 0.05.

**Table 5:** The complement proteins correlated with PSD and VFI values.

Gene Symbol	Description	PSD Glaucoma		VFI Glaucoma	
		Correlation Coefficient (R)	*p*-Value	Correlation Coefficient (R)	*p*-Value
C5	Complement C5	0.47	0.002 *	−0.31	0.125
C7	Complement component C7	0.43	0.006 *	−0.64	0.0006 *
C1R	Complement C1r subcomponent	0.39	0.014 *	−0.52	0.008 *
C4B	Complement C4B	0.38	0.019 *	−0.46	0.020 *
C8A	Complement component C8 alpha chain	0.37	0.020 *	−0.29	0.162
F2	Prothrombin	0.36	0.024 *	−0.49	0.014 *
C4A	Complement C4-A	0.33	0.038 *	−0.44	0.027 *
C9	Complement component C9	0.30	0.063	−0.44	0.027 *
C8G	Complement component C8 gamma chain	0.24	0.143	−0.54	0.006 *

PSD: pattern standard deviation; VFI: visual field index; * *p* < 0.05.

**Table 6:** The complement proteins correlated with HRT parameters.

Gene Symbol	Cup Area (mm^2^)		Cup Volume (mm^3^)		Maximum Cup Depth (mm)		Mean Cup Depth (mm)		Disc Area (mm^2^)		CD Area Ratio	
	Corr. Coeff. (R)	*p*-Value	Corr. Coeff. (R)	*p*-Value	Corr. Coeff. (R)	*p*-Value	Corr. Coeff. (R)	*p*-Value	Corr. Coeff. (R)	*p*-Value	Corr. Coeff. (R)	*p*-Value
CFI	−0.33	6 × 10^−5^ *	−0.33	5 × 10^−5^ *	−0.27	0.001 *	−0.23	0.005 *	−0.19	0.020 *	−0.28	0.0004 *
VTN	−0.26	0.001 *	−0.29	0.0005 *	−0.22	0.009 *	−0.15	0.061	−0.19	0.021 *	−0.19	0.022 *
CLU	−0.26	0.002 *	−0.25	0.002 *	−0.15	0.066	−0.13	0.121	−0.09	0.291	−0.21	0.010 *
C3	−0.22	0.007 *	−0.26	0.002 *	−0.22	0.007 *	−0.19	0.022 *	−0.17	0.045 *	−0.19	0.022 *
CFH	−0.20	0.015 *	−0.23	0.005 *	−0.22	0.008 *	−0.20	0.014 *	−0.14	0.105	−0.21	0.009 *
CFHR1	−0.17	0.036 *	−0.20	0.018 *	−0.18	0.030 *	−0.17	0.032 *	−0.18	0.034 *	−0.16	0.049 *
C4B	−0.15	0.074	−0.19	0.023 *	−0.15	0.074	−0.15	0.062	−0.09	0.307	−0.12	0.143
C2	−0.15	0.065	−0.16	0.049 *	−0.13	0.116	−0.13	0.116	−0.11	0.188	−0.12	0.146
F2	−0.14	0.093	−0.18	0.034 *	−0.20	0.015 *	−0.16	0.054	−0.08	0.358	−0.10	0.213
C5	−0.08	0.358	−0.11	0.194	−0.17	0.038 *	−0.13	0.102	−0.14	0.083	−0.05	0.533
C8B	−0.08	0.360	−0.11	0.171	−0.17	0.044 *	−0.13	0.111	−0.07	0.395	−0.05	0.504

HRT: Heidelberg Retinal Tomography; CD: cup-to-disc; * *p* < 0.05.

## Data Availability

All raw data are available in the Supplementary Materials.

## Supplementary Materials

The following supporting information can be downloaded at https://www.mdpi.com/article/10.3390/jpm13091400/s1, Supplementary File S1: Patient sample information and associated phenotypic data. Supplementary File S2: Normalized protein levels.
